# Knowledge, attitudes and practices of psychiatrists in India regarding sudden unexpected death in epilepsy (SUDEP) and seizure-related harm

**DOI:** 10.1016/j.ebr.2024.100686

**Published:** 2024-06-20

**Authors:** Surobhi Chatterjee, Shivangini Singh, Sujita Kumar Kar, Rohit Shankar

**Affiliations:** a Department of Psychiatry, King George’s Medical University, Lucknow 226003, Uttar Pradesh, India; b Cornwall Intellectual Disability Equitable Research (CIDER), University of Plymouth Peninsula School of Medicine, Truro, UK; c Cornwall Intellectual Disability Equitable Research (CIDER) Cornwall Partnership NHS Foundation Trust, Truro, UK

**Keywords:** Psychiatrists, Epilepsy harm, Lower-middle income countries, Counselling, SUDEP

## Abstract

Sudden unexpected death in Epilepsy (SUDEP) is one of the leading causes of death in people with epilepsy (PWE). Awareness and taking adequate preventive measures are pivotal to reducing SUDEP. Nearly 80% of PWE live in lower-middle-income countries (LMICs) such as India where for many, epilepsy management is by psychiatrists.

To evaluate the knowledge, attitude and practices of Indian psychiatrists on SUDEP and seizure risk.

A cross-sectional online survey of 12 Likert response questions using validated themes, was circulated among Indian Psychiatric Society members. Non-discriminatory exponential snowballing technique leading to convenience non-probability sampling was used. The inquiry involved SUDEP-related topics including the need for and importance of counselling. Descriptive statistics and the chi-square test were used for analysis.

The psychiatrists responding (n = 134) were likely to be males (72.4 %), urban (94 %) and affiliated to academic institutions (76.1 %). Nearly all saw PWE monthly with over half (54 %) seeing more than 10. Nearly two-third (64.17 %) did not counsel PWE regarding SUDEP, due to fear of raising concerns in caregivers/family (33.3 %), patients (38.9 %) or lack of time (35.6 %), though 37 % had lost patients due to SUDEP. Over two-third (66.7 %) agreed risk counselling was important. Barriers included fear of raising concerns, limited time, and training. A strong need for national SUDEP guidelines (89 %) and suitable training (75.4 %) was expressed.

Though epilepsy care is provided by a considerable number of psychiatrists, there is a poor understanding of SUDEP. Enhancing the awareness and understanding of SUDEP is likely to enhance epilepsy care.

## Highlights

• 80 % people with epilepsy (PWE) globally are in Lower Middle-Income Countries (LMIC).
• There is little SUDEP research or literature from India which has 12 million PWE.
• In LMICs like India, psychiatrists might be the sole professionals managing PWE.
• This study explores attitudes, challenges and needs of Indian psychiatrists to SUDEP.
• Patient counselling in SUDEP is a significant gap among psychiatrists in India.

## 1 Introduction

Sudden Unexpected Death in Epilepsy (SUDEP) is defined as a “*sudden, unexpected, witnessed or unwitnessed, nontraumatic and non-drowning death, occurring in benign circumstances, in an individual with Epilepsy, with or without evidence for a seizure after excluding status epilepticus, in which post-mortem examination does not reveal a cause of death*” [1]. SUDEP is the most important direct cause of death in epilepsy [1]. The American Association of Neurology reported that the risk of SUDEP is approximately 1 in 4,500 in children with epilepsy, which increases to 1 in 1,000 in adulthood [2].

Recent research into SUDEP has shown that there are modifiable and non-modifiable risk factors [3], [4]. Modifiable risk factors include reduction in frequency of bilateral generalised seizures, compliance with anti-seizure medication (ASMs), nocturnal monitoring and possible social-environmental factors which may influence seizure control [5]. Increasingly, the recognition of these risk factors is lending itself to the need to make people with epilepsy more aware of their individualised risk of SUDEP [6], [7], [8], [9]. Thus, improved communication understanding the person with epilepsy’s personalised risk and counselling on potential areas to mitigate seizure related harm and SUDEP is becoming imperative to routine epilepsy practice in most if not all developed countries [10].

The knowledge, attitudes, and practices of neurologists worldwide regarding SUDEP vary significantly. Since 2004, the National Institute of Clinical Excellence (NICE) has issued guidelines stating that every person with epilepsy must be informed about SUDEP and its risks by their clinician [11]. However, the implementation of these guidelines is varied [12].

### 1.1 Geographical variations in discussing SUDEP and seizure related risk

There are marked inter-country variations reported by neurologists in the last decade in the frequency of SUDEP discussions. In Eastern Mediterranean 1.7 %, Italy 1.8 %, Austria/Germany/Switzerland 2.7 % in North America (USA and Canada) 12.2 %, 20 % in the UK and 25 % in Saudi Arabia reported discussing SUDEP with all their patients [13], [14], [15], [16], [17], [18], [19]. As much as 60–65 % of neurologists in Saudi Arabia, Italy and the UK discussed SUDEP with very few patients and 7–25 % of them never had any conversation regarding SUDEP at all [14], [18], [19].

In a tertiary hospital in Uganda, 85 % of Physicians did not provide any information regarding SUDEP to their patients [20]. In the largest study done to date on 1123 neurologists from 27 countries, 41.5 % of them reported that they rarely had discussions on the risk of SUDEP with patients and their caregivers [21]. Though no randomised studies exist it is hoped that discussions of SUDEP and it’s risk factors can lead to it’s mitigation and reduction [22]. It also leads to an underestimate of SUDEP deaths by a lack of recognition of it by clinicians and families. It is worth highlighting how poor and varied SUDEP evaluation is globally due to varied legal, coronial, familial, cultural, religious and autopsy-related factors [23]. A study obtaining feedback from 77 countries outlined that only 13 % had confidence in reporting SUDEP as a cause of death. Two-thirds of the countries had not undertaken any research or audits on SUDEP or seizure harm [23].

### 1.2 Whose role is it to counsel SUDEP and seizure related risk matters?

To address the varied needs of an individual patient with epilepsy, a multidisciplinary team is usually required [24]. This team may include adult and paediatric neurologists, paediatricians, child psychologists, psychiatrists, mental health care nurses, and social workers [11]. However, most of the studies conducted to date only evaluate the perspectives of adult and paediatric neurologists [12]. There is presently a lack of clarity on whose role it is to give SUDEP-related information and risk management advice in a multi-disciplinary team especially for vulnerable and higher risk populations, as it is increasing recognised that a single conversation of SUDEP is rarely helpful [25], [26].

### 1.3 Epilepsy risk and Low-middle-income countries

Around 80 % of people with epilepsy live in low −middle-income countries (LMICs) which are resource-deprived and have very few neurologists or indeed specialists in epilepsy [27]. There is a significant psychiatric burden particularly depression and anxiety in people with epilepsy who are thus likely to see psychiatrists, particularly in LMICs [27], [28], [29]. Further, there is evidence suggesting psychiatric and psycho-social issues are risk factors for seizure-related harm, can lower the threshold for SUDEP and can be mitigated by suitable person-centred communication of those risks [24], [30], [31], [32]. Hence, there is a need for a unified clinical approach to tackling SUDEP and seizure harm.

Other than for the UK there has been minimal published research to assess the understanding, mindset, and habits of psychiatrists regarding the risk of seizures and SUDEP [33]. This is particularly important in LMICs where a psychiatrist might be the only professional involved with the person with epilepsy [34]. Recent studies conducted in India highlighted the importance of discussing SUDEP, resulting in an enhancement in medication adherence and safety practices [35], [36].

### 1.4 Epilepsy and India

It is estimated that there are over 12 million people with epilepsy in India [37]. There are however less than 2000 neurologists in India leading to a large treatment gap and medical need [38]. Further, lack of awareness of risks in both patients and general clinicians, affordability, and availability of ASMs, cultural beliefs and stigma contribute [38]. It is estimated that at least a million people have medically refractory epilepsy with a significant majority being eligible for surgery [39]. However, only around 500 epilepsy surgeries occur countrywide [40]. Refractory epilepsy is a significant recognised risk factor for SUDEP [11]. Due to the bidirectional relationship between epilepsy and mental illness, neurology, particularly epilepsy is a core topic of the psychiatry curriculum for higher training in India [41].

This study aims to understand the current awareness among psychiatrists regarding SUDEP in India. The study’s primary objective was to evaluate the knowledge, attitude, and practices of Indian psychiatrists concerning counselling on SUDEP and seizure risk.

## 2 Methods

### 2.1 Study design and participants

The study followed the Checklist for Reporting Results of Internet E-Surveys (CHERRIES) along with the STROBE checklist for reporting the cross-sectional study (Supplementary Information 1).

It was conducted in a tertiary care teaching hospitals, private clinics, and speciality clinics within district hospitals across India. The participants included psychiatrists and psychiatry trainees registered with the Indian Psychiatric Society (IPS) and practicing across India. Respondents would have been included after meeting the selection criteria, which included having a minimum of a medical (MBBS) degree and undergoing psychiatry postgraduate training or completed psychiatry training and currently practicing psychiatry in India, and consenting to participate.

### 2.2 Survey tool

The evidence-based SUDEP clinicians interview tool which provides ten essential questions was utilised as the primary framework for the survey [4]. The survey explored socio-demographic factors such as age, gender, years of practice, and location of practice, as well as knowledge of side effects of antiepileptic medications and patient compliance. Additionally, the survey included eight questions assessing knowledge, attitude, and practices regarding SUDEP counselling and risk factors.

### 2.3 Ethics

The study was reviewed by the Ethics Committee of King George’s Medical University, Lucknow, India and full ethics approval was received (Reference number: 116th ECM IIA/P18, dated −6–06-2022) (Supplementary Information 2). Completion of the survey was regarded as consent to participate in the study and was explicitly mentioned as such.

### 2.4 Statistical analysis

The survey forms were distributed through an online Google survey form using an exponential snowballing technique and employed convenience sampling. The survey link was open between 25-06-2022 to 17-01-2023. Two reminders were given to the contacts for their possible participation in the survey. IBM SPSS Version 23.0 was used to analyse data. Descriptive statistics were represented as mean and standard deviation and the chi-square test was used to compare categorical data. A word-cloud analysis to present the common risk factors of SUDEP was done.

## 3 Results

A total of 134 psychiatrists from India, participated in the study of which the majority (72.4 %) were males and practicing in urban areas (94 %). The majority of the participants were working in academic institutions (76.1 %) and holding an academic position (41.8 %). Majority (56 %) of the participants were seeing more than 100 patients generally in a month. Nearly all (99.6 %) told of seeing one or more people with epilepsy monthly. Over half of the respondents (54 %) told of seeing on average greater than 10 people with epilepsy a month. Just over a third (35.1 %) of respondents were trainees (post basic medical degree) completing their psychiatric higher training. Six percent of the participants informed of taking part in any training related to epilepsy. The characteristics of the participants is described in Table 1.

**Table 1:** Characteristics of the participants took part in the survey.

Age in years (Mean ± SD)	38.07 ± 10.57
Gender	(134)
Male	97 (72.4 %)
Female	37 (27.6 %)
Prefer not to say	0
Location of practice	(1 3 4)
Rural	8 (6 %)
Urban	126 (94 %)
Treatment setting	(1 3 4)
Academic institution	102 (76.1 %)
Private hospital	13 (10.4 %)
District hospital	8 (5.97 %)
Office based	3 (2.2 %)
Others	8 (5.97 %)
Number of years in clinical practice (Mean ± SD)	9.5 ± 9.94
Current position at workplace	(1 3 4)
Academic position	56 (41.8 %)
Non-academic position	10 (7.5 %)
Private practitioner	15 (11.5 %)
Post doctoral fellow in psychiatry	3 (2.2 %)
Pursuing psychiatry course	47 (35.1 %)
Other	3 ((2.2 %)
Approximate number of patients seen in a month	(1 3 4)
Less than 10	8 (5.97 %)
11 – 25 patients	5 (3.7 %)
26 – 50 patients	18 (13.4 %)
51 – 100 patients	32 (23.9 %)
More than 100 patients	75 (56 %)
Approximate number of patients with epilepsy seen in a month	(1 3 4)
None	1 (1.34 %)
Less than 10	59 (44 %)
11 – 25 patients	44 (32.8 %)
26 – 50 patients	11 (8.2 %)
51 – 100 patients	15 (11.2 %)
More than 100 patients	3 (2.2 %)
Any training courses in Epilepsy received	(1 3 4)
Yes	8 (6 %)
No	126 (94 %)
Mode of training	(8)
Online	1 (12.5 %)
Offline	7 (87.5 %)
Type of training session	(8)
Didactic sessions	4 (50 %)
Hands-on-training	4 (50 %)
Training help in improving practice	(8)
Yes	5 (67.5 %)
No	1 (12.5 %)
Not sure	2 (25 %)

The majority (91 %) of the participants had a previous engagement in counselling of people with epilepsy including those patients who had co-morbidities such as intellectual disability or psychiatric disorders. More than 75 % of the participants particularly felt a need of training to deal with communication with people with epilepsy. Approximately 36 % of the participating psychiatrists told of counselled their people with epilepsy or their parents/caregivers about the risk of SUDEP.

Over a third (36.3 %) of participants report losing one to five patients to SUDEP with 10 % being “not sure”.

Just under half of participants (46.6 %) discuss SUDEP once the diagnosis of epilepsy is made and just over a third (37.9 %) when epilepsy becomes refractory. The commonest instances when the participants counsel their patients and carers about SUDEP are when they recognise epilepsy to be refractory (65.4 %), identify treatment non-adherence (55.8 %) or there is the presence of bilateral generalized tonic-clonic seizures (48.1 %). The common reasons for not doing SUDEP counselling in all people with epilepsy are raising concerns in caregivers/family (38.9 %), lack of time (35.6 %) and raising concerns in the patient (33.3 %). While treating the paediatric population with epilepsy, the risk of SUDEP is only discussed with their parents or caregivers by just over half the participants (52.5 %).

Four-fifth (80.3 %) of the participants routinely counselled about other seizure risks in daily life activities such as drowning, falling from heights, and working on rotating machines, to all their people with epilepsy or their caregivers. About a third (35 %) of the psychiatrists or psychiatry trainees educate “majority to all” of the people with epilepsy or their carers about the risk of suicidal ideation associated with antiseizure medications. Nearly three-quarter (73 %) of participants educate people with epilepsy on driving restrictions to “majority to all” people with epilepsy or their caregivers. Two-third of the participants (66.7 %) agree that all the risks of a disease or a therapy should be discussed with the patient. Among the participants, nearly all (92.5 %) agreed that it would be helpful to have specific regulations on a discussion of risks and their frequency.

**Fig. 1:**
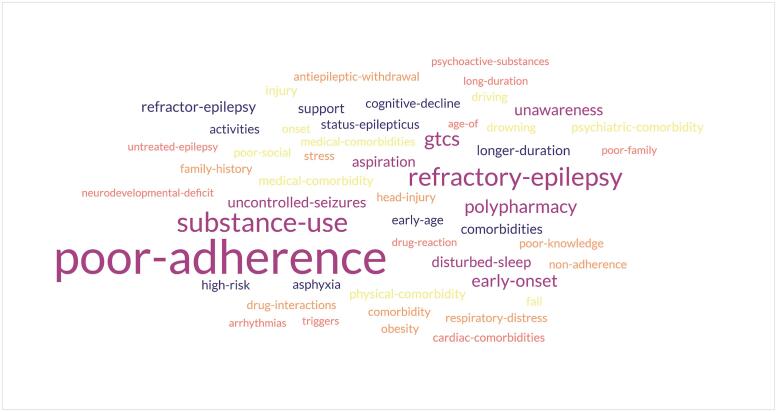
Participant reported risk factors of SUDEP.

Table 2 summarizes the awareness of participants about SUDEP and their extent of engagement in epilepsy care. Fig. 1 displays the common risk factors of SUDEP as reported by the participants. Poor adherence to treatment is reported as the most common risk factor of SUDEP. Other major risk factors of SUDEP reported by the participants are refractory epilepsy and substance use.

**Table 2:** Awareness of participants about SUDEP and their engagement in epilepsy care.

Have you ever counselled patients with Epilepsy?	(134)
Yes	122 (91 %)
No	8 (6 %)
Not sure	4 (3 %)
Have you ever counselled patients with Epilepsy having psychiatric comorbidities or intellectual disability?	(1 3 4)
Yes	123 (91.8 %)
No	8 (6 %)
Not sure	3 (2.2 %)
Do you think you need training to deal with these patients?	(1 3 4)
Yes	75.4 %
No	14.2 %
Not sure	10.4 %
How many patients with epilepsy/parents/caregivers do you counsel about the risk of SUDEP?	(1 3 4)
All of my patients (>90 %)	12 (9 %)
Majority of my patients (50–90 %)	36 (26.9 %)
Minority of my patients (10–49 %)	17 (12.7 %)
Very few patients (1–9 %)	32 (23.9 %)
None of my patients (0 %)	37 (27.6 %)
Which clinical factors lead you to discuss the SUDEP-risk?	(1 0 4)
Refractory course of disease	68 (65.4 %)
Generalized tonic-clonic seizures	50 (48.1 %)
Polytherapy with AEDs	49 (47.1 %)
While recruiting a person with epilepsy to a research study	0
Before epilepsy surgery	3 (2.9 %)
Non-compliance of patient	58 (55.8 %)
Patients demands information	29 (27.9 %)
Patient’s capacity of understanding	32 (30.8 %)
Other	0
At what time do you discuss the risk of SUDEP with your patient?	(1 0 3)
When epilepsy is diagnosed	48 (46.6 %)
Soon after the diagnosis in the first 1–2 years	12 (11.7 %)
When the disease takes a refractory course	39 (37.9 %)
Other	4 (3.9 %)
What do you expect from counselling about the risk of SUDEP?	(1 0 3)
Effective prevention of SUDEP cases	40 (38.8 %)
Improved compliance of patients,	53 (51.5 %)
Improved supervision at night	2 (1.9 %)
Fulfilment of medical duty to discuss the risks	8 (7.8 %)
Why do you counsel none or some patients about SUDEP?	(90)
Lack of consequences	8 (8.9 %)
Raising concerns in the patient	30 (33.3 %)
Raising concerns in caregivers/family	35 (38.9 %)
Lack of time	32 (35.6 %)
Patient is not at high risk for SUDEP	26 (28.9 %)
No known preventive measures against SUDEP	12 (13.3 %)
Poor benefit-risk ratio	6 (6.7 %)
Fear of the emotional reaction of the patient	20 (22.2 %)
With whom do you discuss the risks when treating a child or an adolescent?	(99)
Only with the parents or caregivers	52 (52.5 %)
Both Parents or caregivers and child/adolescent	46 (46.5 %)
Primarily parents adolescents depending on the level of their intellectual ability	1 (1 %)
Did any of your patients die from SUDEP?	(1 0 2)
Yes	37 (36.3 %)
a. 1–5 patients.	37 (100 %)
b. 5–10 patients.	−
c. more than 10 patients	−
Cannot say for sure.	10 (9.8 %)
No, none of my patients.	55 (53.9 %)
How many patients with epilepsy or parents/caregivers do you counsel about risks in daily life activities such as drowning, falling from heights, working on rotating machines	(1 2 7)
All of my patients (>90 %)	70 (55.1 %)
Majority of my patients (50–90 %)	32 (25.2 %)
Minority of my patients (10–49 %)	7 (5.5 %)
Very few patients (1–9 %)	7 (5.5 %)
None of my patients	11 (8.7 %)
How many patients with epilepsy or parents/caregivers do you counsel about suicidal ideations on anti-seizure medication?	(1 2 6)
All of my patients (>90 %)	15 (11.9 %)
Majority of my patients (50–90 %)	29 (23 %)
Minority of my patients (10–49 %)	23 (18.3 %)
Very few patients (1–9 %)	31 (24.6 %)
None of my patients	28 (22.2 %)
How many patients with epilepsy, who are physically and mentally capable of driving, do you counsel about driving restrictions?	(1 2 6)
All of my patients (>90 %)	60 (47.6 %)
Majority of my patients (50–90 %)	32 (25.4 %)
Minority of my patients (10–49 %)	14 (11.1 %)
Very few patients (1–9 %)	17 (13.5 %)
None of my patients	3 (2.4 %)
It is my opinion, that all risks of a disease or a therapy should be discussed with the patient-	(1 2 6)
Yes, all risks should be discussed	84 (66.7 %)
No only frequent risks	19 (15.1 %)
Only risks that cause great damage	11 (8.7 %)
Only preventable risks	7 (55.5 %)
Only when resulting in consequences in action	1 (0.8 %)
I ask the patient how detailed risks should be discussed.	4 (3.2 %)
Would it be helpful to have specification regulations at which frequency risks should be discussed with a patient?	(1 3 4)
Yes	127 (92.5 %)
As clinical practical guidelines of the psychiatric/ neurological societies	113 (89 %)
As statutory specifications.	14 (10.2 %)
No	7 (7.5 %)

## 4 Discussion

Sudden unexpected death is higher (at least 20 times) among people with epilepsy than general population [24]. The majority of the studies related to SUDEP awareness of patients, their caregivers and their clinicians have emerged in economically developed countries usually involving neurologists or general care physicians [12], [23]. Psychiatrists perspective about creating SUDEP awareness is less studied [33]. Psychiatrists also provide care to people with epilepsy especially in LMICs and epilepsy is a comorbidity with many psychiatric disorders. Hence, understanding about SUDEP and informing patients and their caregivers about it is of utmost importance for psychiatrists.

Nearly two thirds of the participants counselled none or very few people with epilepsy they see on SUDEP. Similar findings are seen across the globe from different regions [13], [14], [15], [16], [17], [18], [19], [20], [21]. Table 3 provides a country wide comparison. It has been observed that those with a fellowship degree in epilepsy, are more likely to counsel people with epilepsy about SUDEP [21]. So, having specialized training may be more impactful in facilitating comprehensive epilepsy care with enhanced counselling services. Evidence from both the two studies done in Indian settings suggests that when informed of SUDEP there is a significant improvement in patient behaviour and seizure outcomes [35], [36].

**Table 3:** Examples of SUDEP awareness across the globe.

Country	Author	Discussed about SUDEP
USA	Friedman et al., 2014	6.8 % discussed it regularly
Italy	Galli et al., 2018	16.2 % stated that all should be counselled
U.K.	Galli et al., 2017	2 % counselled all patients
U.K.	Gayatri et al., 2010	20 % counselled all patients
Saudi Arabia	Hakami and Hakami, 2021	25 % discussed it regularly
Middle Eastern Countries	Saleh et al., 2022	1.5 % discussed with most patients
Austria, Germany, Switzerland	Strzelczyk et al., 2016	2.7 % counselled all patients

The findings of nearly two-thirds not or minimally counselling people with epilepsy on SUDEP appears counterintuitive to the finding that most participants agreed that it is essential to discuss all risks associated with a disease or therapy with patients, highlighting the importance of open and comprehensive communication. The challenge appears to be the lack of confidence and ability to give sensitive meaningful person-centred communication on difficult topics such as SUDEP [32]. Some of the barriers to counselling on SUDEP as identified in our study include perceived concerns from caregivers and families, time constraints, and patients’ own fears which have been proven to be broadly unjustified in current literature [24], [26], [30], [31], [32]. Addressing these obstacles is crucial to ensure that patients receive comprehensive care, education and can self-empower themselves. Researchers have developed an evidence-based checklist of major risk factors of SUDEP, which can be used for the prevention of mortality associated with epilepsy in developed Western countries [30], [31].

Less than half of the participants told of those whom they wish to counsel being done so at the time of diagnosis and a third when the epilepsy is refractory. Evidence suggests that by the time healthcare personnel counsel people with epilepsy and their carers about SUDEP, a sizable number of patients reach to a stage of refractory epilepsy [42]. It is thus important to do the SUDEP counselling at an appropriate time. As suggested by the participants in this study, there is still room for improvement by having earlier and more frequent conversations [43].

Evidence suggests that history of bilateral generalized tonic-clonic seizure (GTCS) during the past year is associated with a multi-fold increased risk of developing SUDEP than those having non-generalized tonic-clonic seizures [24]. Similarly, nocturnal GTCS and medication non-adherence are associated with a higher risk of SUDEP [24]. Though GTCS is an important risk factor of SUDEP, in our study less than half of the participants (48 %) discuss about SUDEP in people with epilepsy with GTCS. Further, it is concerning that while GTCS did feature overall as a risk factor, the study cohort did not identify GTCS, or nocturnal seizures in their top three common risk factors, though medication adherence was (Fig. 1).

Our study also found that participants were more proactive in counselling patients about other safety aspects of epilepsy management. The finding that a third of the study cohort would counsel all their patients with epilepsy about suicidal risk with anti-seizure medication is interesting. Overall, the available literature does not support an obvious causal relationship between suicide and anti-seizure medication though some ASMs are recognised to have negative psychotropic properties which can for some people with epilepsy worsen mood and behaviour [44], [45].

Another issue is the amount of exposure psychiatric trainees in India have to neurology in general and epilepsy in particular. The national postgraduate psychiatry curriculum advocates for 2 to 6 months structured training in neurology for psychiatric post graduate trainees across all medical institutions offering psychiatric training including competency in basic epilepsy management [46]. The delivery of this curriculum is varied across the country and recognised to be in decline [47].

### 4.1 Limitations

The results of this survey should be viewed in the context of the responder rate that is low compared to the total number of potential responders though numerous avenues for dissemination were used. It is not known if the same psychiatrists who had reported SUDEP deaths were the ones that counselled for this. Further, as described inspite of the curriculum endorsing training in epilepsy and SUDEP matters it is unclear how much awareness the respondents would have of this issue. In addition, the survey failed to inquire into the years of clinical experience individual respondents had. Further, given over proportion of responses from urban practice, males and academic institutions the results of the survey are not generalisable and would need to be interpreted with caution.

Comorbidities with epilepsy such as psychiatric comorbidities are not evaluated as someone with mental illness and epilepsy may not be a right candidate for explanation of SUDEP. Social circumstances in those with epilepsy such as homelessness and other issues such as confidentiality in married women may restrict the time psychiatrists in India may need to get to this sensitive topic. Further, we do not know, if these psychiatrists refer the patients to neurology/ physician colleagues in which case the latter may explain about this condition to the patients, though it is good medical practice to check or re-discuss it. Finally, diagnosis of type of epilepsy and age group of patients seen is not known. Having training alone may not be sufficient. The study does not address the massively low doctor patient ratio in India, which may pose a challenge to spend structured time and identify the risk factors for SUDEP. Thus, having a structured proforma like the SUDEP and Seizure Safety Checklist validated for use in India might be useful [30], [31].

## 5 Conclusion

There are around 12 million people with epilepsy in India [37]. It is recognised that SUDEP would be a major cause of death in this population [48]. However, till date there is little research or awareness of SUDEP as there are only two studies from India till date which have looked at SUDEP related matters [35], [49]. While this suggests a significant gap in reducing preventable mortality in epilepsy it is consistent with the challenges of many other LMICs on SUDEP matters [23].

A Population based study in India has shown the prevalence of psychiatric illness in people with epilepsy to be as high as 52 % [50]. This thus justifies the need for psychiatrists particularly in LMICs to be epilepsy and SUDEP aware.

This is the first study in India and possibly in South-East Asia which explores the awareness and attitudes of a clinical group involved in epilepsy to SUDEP. It is also likely to be the first study globally exploring awareness and attitudes of psychiatrists to SUDEP. The findings of this study have highlighted the importance of enhancing the level of engagement between psychiatrists and psychiatry trainees in counselling people with epilepsy. A considerable number of participants have acknowledged the need for additional training, particularly in managing people with epilepsy who have co-existing psychiatric comorbidities or intellectual disabilities. Moreover, there is a clear requirement to improve counselling practices for people with epilepsy and their caregivers regarding different aspects of epilepsy management. Our recommendations for improving SUDEP counselling for people with epilepsy in India are as follows:

1. Develop risk counselling programs for all clinicians involved in epilepsy including psychiatrists and trainees in LMICs with a focus on SUDEP.

2. Ensure that SUDEP counselling is integrated into routine epilepsy care in general and psychiatric training in particular globally.

3. Discuss all relevant risks with people with epilepsy, particularly SUDEP.

4. Establish guidelines and validate counselling tools such as the SUDEP and seizure safety Checklist in Indian setting for discussing SUDEP and epilepsy risks in a person-centred manner.

Projects to develop and implement these recommendations could be similar to other international programmes such as those for primary care as devised by the ILAE [51]. By implementing these recommendations, healthcare providers can offer better care and support for people with epilepsy, which can lead to better health outcomes while reducing the negative outcomes associated with epilepsy.

## Funding

This research did not receive any specific grant from funding agencies in the public, commercial, or not-for-profit sectors.

## Author contributions

All authors satisfy the ICMJE guidance by substantially contributing to the design, analysis and interpretation of the work, drafting of the manuscript, final approval of the manuscript and all agree to be accountable for all aspects of the work in ensuring that questions related to the accuracy or integrity of any part of the work is appropriately investigated and resolved.

## CRediT authorship contribution statement

**Surobhi Chatterjee:** Writing – original draft, Formal analysis, Data curation. **Shivangini Singh:** Writing – original draft, Investigation, Data curation. **Sujita Kumar Kar:** Writing – review & editing, Supervision, Project administration, Methodology, Investigation, Formal analysis, Data curation. **Rohit Shankar:** Writing – review & editing, Visualization, Validation, Supervision, Resources, Project administration, Methodology, Conceptualization.

## Declaration of competing interest

The authors declare the following financial interests/personal relationships which may be considered as potential competing interests: RS has received institutional and research support from LivaNova, UCB, Eisai, Veriton Pharma, Bial, Angelini, UnEEG and Jazz/GW pharma outside the submitted work. He holds grants from NIHR AI, SBRI and other funding bodies all outside this work. No other authors has any declared conflict of interest to this paper.

## Figures and Tables

**Supplementary Fig. 1:**
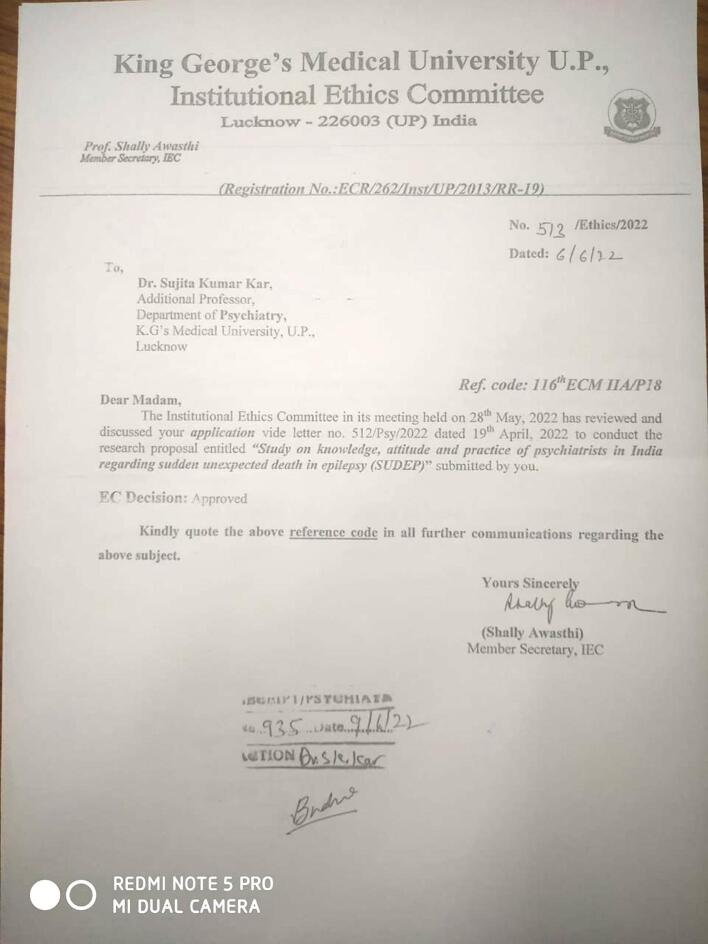


## Data Availability

The data that support the findings of this study are available from the corresponding author upon reasonable request.
